# Evaluation of the Ca37 Monoclonal Antibody Targeting Alcohol Dehydrogenase Against *Candidozyma auris* (*Candida auris*) In Vitro and In Vivo

**DOI:** 10.3390/jof11120864

**Published:** 2025-12-05

**Authors:** Oier Rodriguez-Erenaga, Maialen Areitio, Lucia Abio-Dorronsoro, Nahia Cazalis-Bereicua, Leire Aparicio-Fernandez, Leire Martin-Souto, Idoia Buldain, Beñat Zaldibar, Aitor Rementeria, Aitziber Antoran, Andoni Ramirez-Garcia

**Affiliations:** 1 MicrobiomicsEHU Research Group, Department of Immunology, Microbiology and Parasitology, University of the Basque Country (UPV/EHU), 48940 Leioa, Spain; oier.rodriguez@ehu.eus (O.R.-E.); maialen.areitio@ehu.eus (M.A.); lucia.abio@ehu.eus (L.A.-D.); nahia.cazalis@ehu.eus (N.C.-B.); leire.aparicio@ehu.eus (L.A.-F.); leire.martin@ehu.eus (L.M.-S.); andoni.ramirez@ehu.eus (A.R.-G.); 2 Section of Immunology, Vetsuisse Faculty, Institute of Experimental Immunology, University of Zurich (UZH), 8057 Zurich, Switzerland; 3 MicrobiomicsEHU Research Group, Department of Immunology, Microbiology and Parasitology, University of the Basque Country (UPV/EHU), 01006 Vitoria-Gasteiz, Spain; idoia.buldain@ehu.eus; 4 CBET Research Group, Department of Zoology and Animal Cell Biology, Faculty of Science and Technology, Research Centre for Experimental Marine Biology and Biotechnology PIE, University of the Basque Country (UPV/EHU), 48940 Leioa, Spain; benat.zaldibar@ehu.eus

**Keywords:** *Candidozyma auris*, *Candida auris*, Ca37 monoclonal antibody, alcohol dehydrogenase, invasive infections, treatment, immune recognition

## Abstract

*Candidozyma auris* (*Candida auris*) is an emerging pathogenic yeast of global concern due to its persistence on abiotic and biotic surfaces and the difficulty of treating the severe infections it causes, which are frequently associated with high mortality rates because of its extensive antifungal resistance. Thus, new therapeutic strategies are urgently needed to complement or replace current antifungal drugs. In this study, we evaluated the efficacy of Ca37, a monoclonal antibody (mAb) targeting the alcohol dehydrogenase (Adh) protein of *Candida albicans*, against *C. auris* both in vitro and in vivo. Protein electrophoresis and Western Blot analyses demonstrated immunoreactivity of Ca37 mAb with *C. auris* total protein and cell wall-associated protein extracts, among which Adh was identified. In vitro, incubation with Ca37 mAb significantly reduced the growth of several *C. auris* strains and enhanced the phagocytic activity of RAW 264.7 murine macrophages. In vivo, Ca37 mAb treatment increased the survival of *Galleria mellonella* larvae. In a murine model of systemic infection, treated mice displayed improved clinical condition, along with a greater number and larger area of immune-associated foci in the kidneys, suggesting enhanced fungal recognition. These findings support the potential of Ca37 mAb as an antifungal immunotherapy, although further studies in murine models are necessary to establish optimal dosing, efficacy, and mechanisms of action.

## 1. Introduction

Since its discovery in Japan in 2009 [1], *Candidozyma auris* (formerly known as *Candida auris*) [2] has emerged as a serious global health concern. In fact, in 2022 the World Health Organization (WHO) included this yeast in the critical group of the fungal priority pathogens list [3]. One of the most alarming traits is its significant multi-drug resistance to antifungal agents, with some strains being resistant to all available antifungal treatments against systemic candidiasis, also known as panresistant clinical isolates [4]. Furthermore, its ability to persist on both abiotic and biotic surfaces facilitates outbreaks in healthcare settings, where it can easily spread among immunocompromised patients [5,6]. Additionally, it is often misidentified in clinical laboratories, delaying accurate diagnosis and treatment [7]. All of these characteristics contribute to the high mortality of hospital-associated infections caused by this yeast, which can reach up to 60% [8].

Currently, there are only four classes of antifungal agents available for treating invasive candidiasis, with resistance observed against each class in *C. auris*. Consequently, there is an urgent need for novel treatments to address infections caused by this species [4]. In this context, monoclonal antibodies (mAbs) constitute a promising therapeutic option for candidiasis and other fungal infections, either on their own or in combination with existing antifungal agents [9].

Previously, our research group developed the IgG1 Ca37 monoclonal antibody against the *Candida albicans* alcohol dehydrogenase 1 (Adh1), a cytosolic and cell wall-associated protein (CWAP) that not only plays a metabolic role but also functions as an antigen and allergen [10]. Moreover, it may even act as an adhesin by binding to fibronectin [10] and serum plasminogen of the host [11]. Indeed, the Ca37 mAb demonstrated antifungal efficacy against *C. albicans* in vitro and in vivo in the *Galleria mellonella* animal model [12].

It is worth highlighting that *C. albicans* Adh has low homology with the human protein [12], but is highly conserved among different species of *Candida* and related genera, which makes it an interesting therapeutic target. In fact, the Ca37 mAb also showed reactivity against *Candida parapsilosis*, *Nakaseomyces glabratus* (formerly *Candida glabrata*), and *C. auris* [12]. Specifically, in the case of the *C. auris* Adh, the homology with *C. albicans* Adh1 protein is 80% as confirmed by BLAST analysis “https://blast.ncbi.nlm.nih.gov/Blast.cgi (accessed on 19 September 2025)”.

Therefore, given the reduced availability of effective treatments against *C. auris* and the potential activity that the Ca37 mAb could also have against this species, the aim of this work was to evaluate the effect of the Ca37 mAb on *C. auris* both in vitro and in vivo. First, we examined the immunoreactivity of Ca37 mAb against *C. auris* cell wall-associated proteins. We then characterised its in vitro activity by assessing fungal growth inhibition after 18 h and the potential opsonisation effect using a murine macrophage cell line. Finally, we evaluated the protective effect of Ca37 mAb in vivo in both *Galleria mellonella* and a murine model of systemic infection.

Altogether, this study provides new insights into the therapeutic potential of Ca37 mAb against *C. auris*, while also highlighting the need for further in vivo investigations to optimise efficacy and safety.

## 2. Materials and Methods

### 2.1. Yeast and RAW 264.7 Cell Line Cultures

Five *C. auris* isolates belonging to the clade III from bloodstream infections were used in this study: four non-aggregative isolates, CECT (Spanish Culture Type Collection) 13225, CJ-194, CJ-195, and CJ-196, and an aggregative one, CECT 13226. These *C. auris* isolates were obtained from an outbreak at La Fe University and Polytechnic Hospital (Valencia, Spain) and were provided by Dr. Javier Pemán.

All yeast strains were cryopreserved at −80 °C and cultured on Sabouraud Dextrose Agar (SDA) (VWR, Radnor, PA, USA) at 37 °C for 24 h prior to use. To obtain *C. auris* cells, the fungi were suspended in Phosphate-Buffered Saline (PBS) (Corning Inc., Corning, NY, USA). The cell density was adjusted using a Bürker counting chamber to inoculate 10^5^ yeast cells/mL in Sabouraud Dextrose Broth (SDB) (VWR) and cells were incubated at 37 °C overnight with shaking at 120 rpm. Finally, the fungal cells were collected by centrifugation (8100× *g* for 3 min), washed twice in PBS and adjusted to the needed concentration.

RAW 264.7 murine macrophage cell line (ATCC, Manassas, MA, USA) was grown in RPMI 1640 supplemented with 10% heat-inactivated Fetal Bovine Serum (VWR), L-glutamine (2 mM) (VWR), penicillin (10,000 U/mL), streptomycin (10 mg/mL) and amphotericin B (25 μg/mL) (Thermo Fisher Scientific, Waltham, MA, USA) at 37 °C, 5% CO_2_ and 95% humidity atmosphere. Cell passages were done when cells reached 80–90% confluence. In addition, cell viability was determined by staining an aliquot with 0.4% Trypan Blue Solution (Merck, Darmstadt, Germany) and counting in a Bürker counting chamber. All assays were performed with cultures showing at least 95% viability.

### 2.2. C. auris Total Protein and CWAP Extraction

Total protein and CWAP were extracted independently from *C. auris* CECT 13225, following the methodologies described by Areitio et al. [13] and Pitarch et al. [14], respectively. For total protein extracts, yeast cells were resuspended in PBS containing 1% β-mercaptoethanol (Merck) and 1% Pharmalyte (Cytiva, Washington, DC, USA). For CWAP, cells were resuspended in lysis buffer (10 mM Tris-HCl, pH 7.4; 1 mM phenylmethylsulfonyl fluoride [PMSF]). In both cases, yeast cells were mixed with 0.5 mm glass beads and disrupted using a MillMix 20 BeadBeater (Tehtnica, Železniki, Slovenia) for 20 min at 30 Hz. The lysates were centrifuged to separate the supernatant and pellet. For total protein extract, the supernatant was collected and stored at −20 °C until use. For CWAP, the pellet was retained and sequentially washed five times with each of the following cold solutions: distilled H_2_O, 5% NaCl, 2% NaCl, 1% NaCl, and 1 mM PMSF. The washed pellet was subsequently treated with SDS extraction buffer (50 mM Tris-HCl, pH 8.0, 0.1 M EDTA, 2% SDS, 10 mM DTT) for 10 min at 100 °C and centrifuged. The resulting supernatant was collected as the CWAP fraction and stored at −20 °C until use.

### 2.3. Detection and Identification by LC-MS/MS of the Immunoreactive Protein Spots

Protein separation by SDS-PAGE was carried out by loading protein samples onto 10% acrylamide gels, which were run at 70 mA, 100 W, and 200 V for 45 min in a Mini-PROTEAN II system (Bio-Rad, Hercules, CA, USA). Page Ruler Plus (Thermo Fisher Scientific) was used as the molecular weight marker. For two-dimensional electrophoresis, the method described by Antoran et al. [12] was applied, using 7 cm Immobiline DryStrip gels pH 3–10 (Cytiva) for isoelectric focusing under the following conditions: rehydration for 12 h, 500 V for 2000 Vhr, 1000 V for 3000 Vhr, 5000 V for 10,000 Vhr, and 5000 V for 40,000 Vhr. Proteins were then separated by SDS-PAGE, and gels were stained with Coomassie Brilliant Blue R250 (Merck) for protein visualisation.

For WB, proteins were transferred to Amersham Hybond-P PVDF membranes (Cytiva) for 1 h at 400 mA using a Trans-Blot Semi-Dry Transfer Cell system (Bio-Rad) with Bjerrum Schafer-Nielsen buffer (0.582% [*w*/*v*] Tris, 0.293% [*w*/*v*] glycine, 20% [*v*/*v*] methanol, pH 9.2). Successful transfer was confirmed by staining with Ponceau Red (0.2% [*w*/*v*] Ponceau Red and 1% [v/v] acetic acid) (Merck). After blocking, the membranes were incubated overnight at 4 °C with the primary Ca37 mAb at a concentration of 60 μg/mL in Tris-Buffered Saline Milk (TBSM; 50 mM Tris-HCl, pH 7.5, 150 mM NaCl, 0.1% [*w*/*v*] Tween 20, and 5% [*w*/*v*] skimmed milk powder; all from Merck). After washing the membrane four times for 5 min each with TBS, the secondary antibody, anti-mouse IgG-HRP conjugate (DC02L, Merck), diluted 1:100,000 in TBSM, was added and incubated for 30 min. The membrane was then washed again with TBS and the proteins of interest were visualised using the ECL Prime chemiluminescence kit (NZYTech, Lisbon, Portugal) in a G: BOX Chemi imaging system (Syngene, Cambridge, UK). All incubation steps were performed at room temperature with constant agitation, unless otherwise specified.

The selected protein spots were manually excised from gels stained with Coomassie Brilliant Blue G250 (Merck) and identified by Liquid Chromatography–Mass Spectrometry (LC-MS/MS) at the University of the Basque Country (UPV/EHU) proteomics service, SGIker, following the method described by Areitio et al. [13], with some modifications. Briefly, LC-MS/MS analyses were performed using Exploris 240 mass spectrometer (Thermo Fisher Scientific), coupled to an EASY-nLC 1200 system (Thermo Fisher Scientific). Peptide sequences were searched against the *C. auris* subset of the UniProt database “https://www.uniprot.org/ (accessed on 6 June 2025)” for protein identification. The mass spectrometry proteomics data have been deposited to the ProteomeXchange Consortium via the PRIDE [15] partner repository with the dataset identifier PXD070229. 

### 2.4. Inhibitory Effect of Ca37 Monoclonal Antibody on Fungal Growth In Vitro

To assess the impact of the Ca37 mAb on *C. auris*, we followed the protocol established by Magliani et al. [16]. *C. auris* CECT 13225 yeast cells were adjusted to a density of 1.5 × 10^3^ cells/mL and incubated with antibody concentrations of 1, 2, 10 and 20 μg/mL and without antibody at 37 °C and 120 rpm for 18 h. To remove antibody aggregates, the mAb was subjected to a thermal-cycling treatment as described by Sadavarte and Ghosh [17], and then filtered prior to use.

Once the optimal concentration (10 μg/mL) was selected, all isolates described in Section 2.1 were tested. To verify the specificity of Ca37 mAb inhibition, an IgG1 isotype control antibody (M5384, Merck) was included and incubated with the yeast cells under the same conditions. Following incubation, three independent replicates of each condition, untreated control, IgG1 isotype control, and Ca37 mAb treatment, were plated on SDA. The plates were incubated at 37 °C for 24 h, and the resulting Colony-Forming Units (CFUs) were counted. Growth in the Ca37 mAb and IgG1 isotype groups was normalised to the untreated control to calculate the percentage of growth.

In addition, a growth curve was performed using *C. auris* CECT 13,255 to characterise the kinetics of the Ca37 mAb. A total of 1.5 × 10^3^ cells/mL were incubated at 37 °C for 40 h. Absorbance was measured at 600 nm using a Synergy TM HT plate reader (BioTek, Winooski, VT, USA) every 10 min after vigorous shaking for one minute. Incubations were carried out in SDB medium under three conditions: fungal cells incubated with PBS (untreated control), 10 µg/mL Ca37 mAb, or 10 µg/mL IgG1 isotype control. In addition, medium-only controls containing PBS, Ca37 mAb, or IgG1 isotype were also included. Absorbance values were converted to *C. auris* log_10_ cells/mL.

### 2.5. In Vitro Phagocytosis Assay

To study the role of the mAb in opsonisation, a phagocytosis assay was conducted in 24-well plates. In each well, a 12 mm diameter coverslip was placed, and on top of it, 10^5^ RAW 264.7 macrophages were seeded in supplemented RPMI 1640. The cells were incubated for 24 h, allowing their number to approximately double. After incubation, the liquid from the wells was removed and co-incubation with *C. auris* CECT 13225 was initiated using the same medium but without amphotericin B. A Multiplicity of Infection (MOI) of 5 was used and 10 μg/mL Ca37 mAb was added to each well of the treatment group, while PBS was added to the untreated control group. After co-incubation periods of 1, 2, and 4 h at 37 °C, 5% CO_2_ and 95% humidity atmosphere, the coverslips were removed, and placed in cold PBS to stop the phagocytosis. Finally, these coverslips were examined under a reverse microscope (Eclipse TE2000-U, Nikon, Tokyo, Japan), and the phagocytosis percentage ([phagocytic macrophages/total macrophages] × 100) and the phagocytic index ([phagocytised yeast/phagocytic macrophages] × 100) were calculated. For each incubation time, three biological replicates were prepared for both the Ca37 mAb and PBS groups, each consisting of three wells. In every well, at least 100 macrophages were counted across five fields of view. 

### 2.6. Galleria mellonella Infection Studies

*Galleria mellonella* sixth-instar larvae (200–250 mg) were obtained from Reptimercado S. COOP (Murcia, Spain). Larvae were kept without food overnight in the dark before use and cleaned with 70% ethanol on the day of the experiment. For inoculation, syringes with a 26G needle (Hamilton, Reno, NV, USA) were used. For optimal infection dose assessment, 10 μL of inoculum were injected into the last left pro-leg of each larva. Three infection doses of *C. auris* CECT 13225, corresponding to 5 × 10^5^, 10^6^ and 5 × 10^6^ cells per larva, were tested. Before the first use, after changing treatments and every six injections the syringe was cleaned with the following products: 10% bleach, 100% ethanol, sterile filtered distilled water, and sterile filtered PBS [18]. Sixteen larvae were used per group, and two negative controls were included in all experiments: non-injected (only cleaned with 70% ethanol) and injected with sterile filtered PBS (PBS control). This approach allowed for the monitoring of natural larval death and death resulting from the injection procedure. After inoculation, larvae were incubated at 37 °C, and mortality was observed daily over 7 days by removing dead larvae. Finally, the inoculated yeasts were plated on SDA in triplicate, allowing CFU counts to determine the initial inoculum density.

Once the optimal infection dose of 5 × 10^6^ cell/larva was established, the effect of Ca37 mAb as treatment was evaluated. For this purpose, each larva from all groups received two injections of 10 μL in the last pro-legs. The first injection involved inoculating *C. auris* in the las left pro-leg, and the additional injection was administered in the last right pro-leg using either PBS, 10 μg/mL Ca37 antibody, or 5 mg/kg micafungin. To achieve the final concentrations in the larvae, the average weight of the group was taken into account. Micafungin dosage was adjusted according to larval weight, while for Ca37 mAb, grams were assumed to equal millilitres, and the stock was prepared accordingly.

Micafungin was chosen as positive control treatment, as it is commonly used against infections caused by *Candida* species. The dose used in this study has been used as a treatment against *C. auris* yeast in mice [19] and even against *Candida* species in *G. mellonella* [20].

### 2.7. Murine Infection Studies

Six-week-old Swiss male and female mice, sourced from Janvier Labs (Le Genest-Saint-Isle, France), were housed and used at the SGIker Animal Facility of the UPV/EHU. They were kept in sterile, filter-aerated cages and had continuous access to food and water. The UPV/EHU Animal Experimentation Ethics Committee approved all experimental protocols (M20/2023/121). In total, 48 immunocompetent mice (24 males and 24 females) were used and divided into four groups: PBS—PBS (uninfected untreated), PBS—Ca37 mAb (uninfected treated), *C. auris*—PBS (infected untreated), and *C. auris*—Ca37 mAb (infected treated). Each group consisted of six female and six male mice, which were split into two groups (three males and three females), to perform two independent experiments.

Mice were anesthetised with an intraperitoneal injection of 100 mg/kg ketamine and 10 mg/kg xylazine. A total of 5 × 10^7^ *C. auris* CECT 13225 cells, suspended in 0.2 mL of PBS, were intravenously injected into the tail vein of each animal in the two infection groups. The inoculation dose of *C. auris* was selected based on a previous study of our research group that demonstrated its ability to consistently establish infection in murine models [13]. The two uninfected groups received 0.2 mL of PBS as the first injection. Additionally, after the first injection and on days three and six, mice were treated with either PBS or the Ca37 mAb. The dose of 10 μg/mL Ca37 mAb was selected based on preliminary data demonstrating its efficacy both in vitro and in vivo (using *G. mellonella*) against *C. albicans* [12] and *C. auris* in this study. The dose for mice was calculated based on the assumption that blood volume represents approximately 6% of body weight [21]. Thus, the Ca37 mAb dose was adjusted to this estimated blood volume of each mouse according to its body weight on the injection day. The *C. auris* inoculum was confirmed by plating and counting serial dilutions of the infection dose on SDA plates. 

Every day, mice were weighed and a wellbeing evaluation was carried out following the approved symptoms scoring table (Table S1). All types of symptoms were studied, although two major groups predominated: physical symptoms (hunched abdomen and ruffled fur) and neurophysiological symptoms (particularly leaning to one side and stereotypies). Eleven days post-infection, the mice were euthanised and brain, lungs, kidneys, spleen, and liver were collected. Each organ was divided into two halves; one half was used for fungal load determination via CFUs counting, and the other half was preserved for histological examination.

For the fungal load assessment, the organs were weighed and homogenised in 1 mL of PBS. A 0.1 mL aliquot of the diluted homogenate was plated in duplicate on SDA plates containing 10 μg/mL chloramphenicol and 25 μg/mL gentamicin (Merck). Plates were incubated at 37 °C, and CFUs were counted after 2–3 days. The limit of detection (LOD) was defined as the lowest microbial density (log CFU/g) required to detect one CFU in the plated volume. Data below the LOD, including zero values, were censored at this limit. Fungal load was calculated as CFU per gram of organ tissue. Log10 transformations were applied to CFU data to normalise the distribution before statistical analysis.

Following dissection, organ samples were fixed in neutral buffered formaldehyde and routinely dehydrated in a graded series of ethanol for subsequent histological examination. Paraffin blocks were obtained from kidney samples, as they were the organs with the highest fungal burden. Five µm thick sections were obtained in a Leica RM 2125RT microtome (Leica Biosystems, Nussloch, Germany) and sections were adhered to previously albumin coated microscopical slides. Slides where then dried overnight at 37 °C and then Haematoxylin–Eosin (H&E) stain was carried out for general observation [22]. For Periodic Acid-Schiff (PAS) staining, sections were dewaxed in xylene and rehydrate in a graded series of ethanol to distilled water. Samples were oxidised with periodic acid (1%) for 10 min and rinse in distilled water. Then, sections were cover with Schiff reagent for 20 min and rinsed in running tap water for 5 min. Nuclei were stained with haematoxylin for counterstain and after differentiation, dehydrated in graded series of ethanol, cleared with xylene and coverslip [23].

To determine the volume density of the granulomatous inflammations observed in kidney samples, the area of the total renal tissue and the area on the inflammations were outlined by hand and determined with the aid of an image analysis software (ImageJ v1.54, National Institute of Health, Bethesda, MA, USA). Then, the volume density was calculated based on the relation between the area of the inflammations and the total area of the renal tissue ([area of the inflammation/total area of renal tissue] × 100).

### 2.8. Statistics

Each experiment was conducted independently three times, except for the *Galleria mellonella* and mouse infection studies. For *G. mellonella*, experiments were repeated twice with n = 32 larvae per group. For the mouse experiments, a total of 48 mice (24 males and 24 females) were used, split into two independent experiments of 24 mice each. Statistical analysis was performed using IBM SPSS Statistics 22 (Professional Statistic, Chicago, IL, USA). Data normality was tested using the Shapiro–Wilk test, and homogeneity of variances was assessed using the Levene’s test. For normally distributed data, analysis was performed using Student’s *t*-test for two-group comparisons, or U-Mann–Whitney for no-normally distributed ones. Statistical analyses comparing survival curves of *G. mellonella* were conducted using GraphPad version 8.0.2, employing the Mantel–Cox test. A significance level of *p* < 0.05 was set for all comparisons.

## 3. Results

### 3.1. Specificity of the Ca37 Monoclonal Antibody Against C. auris CWAP

**Figure 1:**
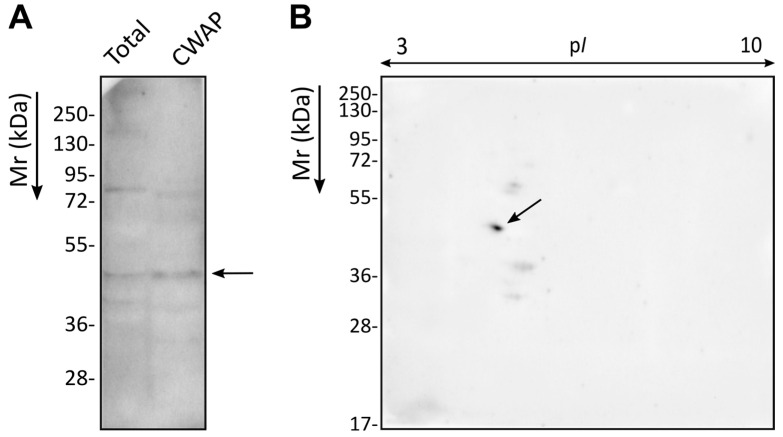
Ca37 monoclonal antibody immunoreactivity towards total and cell wall-associated protein (CWAP) extracts of *Candidozyma auris*. (**A**) Recognition of both total protein and CWAP extracts of *C. auris* by Ca37 mAb using SDS-PAGE followed by WB. (**B**) Recognition of CWAP of *C. auris* by Ca37 mAb using 2D-WB. The arrows point to the most immunoreactive bands and spot. The marked spot in the 2D-WB was manually excised and identified by LC-MS/MS.

To identify the proteins recognised by the Ca37 mAb in *C. auris*, first SDS-PAGE followed by WB was performed. Both *C. auris* total protein and CWAP extracts were analysed, using Ca37 mAb as the primary antibody. The Ca37 mAb showed high immunoreactivity against a protein band with a molecular weight of approximately 49 kDa in both protein extracts (Figure 1A). As our aim was to evaluate Ca37 mAb as a potential therapeutic antibody, we focused on proteins located at the cell surface and thus accessible to recognition. For this reason, the two-dimensional electrophoresis followed by Western blotting (2D-WB) was performed using only the CWAP extract. In this case, the mAb showed high immunoreactivity at molecular weight around 49 kDa and isoelectric point of 4.9–5.1 (p*I*), (Figure 1B), similar to the experimental values previously reported for *C. albicans* Adh1 [12]. This signal corresponded to two very closely spaced spots in the Coomassie-stained gel, which were manually extracted and subjected to identification by LC-MS/MS. In both cases, Adh was detected with a coverage exceeding 40%, although the results showed a mix of several proteins, and Adh was not the most abundant in terms of score or coverage.

### 3.2. Effect of the Ca37 mAb Against C. auris In Vitro

**Figure 2:**
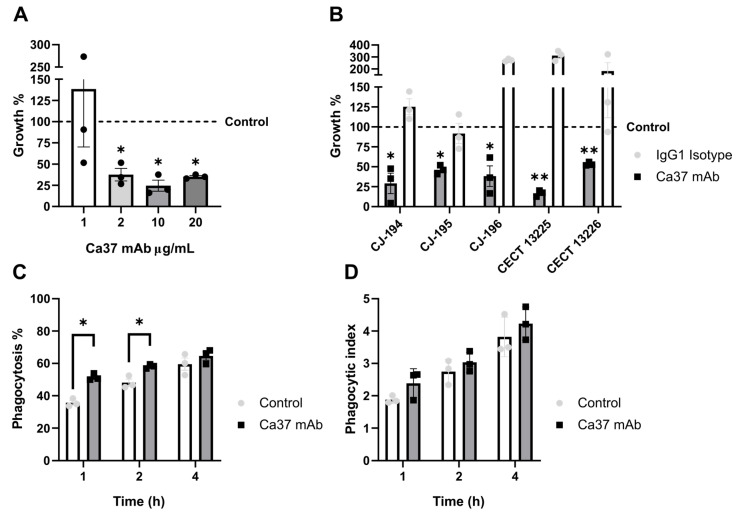
In vitro effect of Ca37 mAb on growth inhibition and on RAW 264.7-mediated opsonisation of *Candidozyma auris*. (**A**) Growth inhibition of the Ca37 mAb at different concentrations (1, 2, 10 and, 20 µg/mL) after 18 h of incubation with *C. auris* CECT 13225, compared to the untreated yeast (dashed line). (**B**) Growth analysis of *C. auris* isolates treated with 10 µg/mL Ca37 mAb and 10 µg/mL IgG1 isotype after 18 h, compared to the untreated yeast (dashed line). (**C**) Phagocytosis percentage of RAW 264.7 macrophages co-incubated with *C. auris* CECT 13225 at an MOI 5 after 1, 2, and 4 h, treated with 10 µg/mL Ca37 mAb or untreated (control). (**D**) Phagocytic index of RAW 264.7 macrophages co-incubated with *C. auris* CECT 13225 at an MOI 5 after 1, 2, and 4 h, treated with 10 µg/mL Ca37 mAb or untreated (control). Data are presented as mean ± SEM. Statistically significant differences in comparison to the untreated control are marked as * *p* < 0.05; ** *p* < 0.01 (two-tailed, unpaired, Student’s *t* test).

To determine the optimal concentration of the Ca37 mAb against *C. auris* CECT 13225, four different concentrations were studied in vitro. Among them, 2, 10, and 20 µg/mL exerted a statistically significant inhibition of growth after 18 h of incubation at 37 °C (Figure 2A). In particular, the 10 µg/mL concentration exhibited the most pronounced inhibitory effect, achieving an inhibition percentage of 75.5% compared to the untreated control. Consequently, this concentration was used for all subsequent experiments. Additionally, a growth curve in SDB at 37 °C was performed using this concentration of Ca37 mAb to assess its effect on *C. auris* growth without the 18 h pre-incubation. No significant growth delay was observed (Figure S1), indicating that the antibody did not inhibit fungal growth under these conditions. 

To further validate this effect, additional four clinical isolates of *C. auris* were also tested and, in all cases, the Ca37 mAb significantly reduced fungal growth compared to the untreated control after 18 h of incubation. Specifically, the percentage of inhibition varied among isolates, ranging from 46.5% to 83% compared to the untreated control (Figure 2B). In those experiments, an IgG1 isotype control was also used as a non-specific antibody control for comparison. The isotype control showed no inhibition and, on the contrary, allowed yeast growth in some strains. Consequently, the inhibition induced by the Ca37 mAb was even more pronounced when compared to the isotype control (Figure 2B). Since CECT 13225 showed the greatest growth reduction with the mAb and also exhibited the highest minimal inhibitory concentration (MIC) to amphotericin B and micafungin among all tested isolates [24], this strain was selected for all subsequent experiments to further analyse the effect of the Ca37 mAb.

Once growth inhibition had been analysed, the opsonisation capacity of the Ca37 mAb was also evaluated. To assess this, *C. auris* yeasts were combined with Ca37 mAb or left untreated (control) immediately before co-incubation with RAW 264.7 macrophages. The phagocytosis percentage and phagocytic index were quantified at 1, 2 and 4 h. The phagocytosis percentage represents the proportion of macrophages that phagocytosed yeasts, whereas the phagocytic index indicates the number of yeasts phagocytosed per phagocytic macrophage. The Ca37 mAb treatment significantly increased the phagocytosis percentage at 1 and 2 h by 44% and 22%, respectively, relative to the untreated group (Figure 2C). It also increased the phagocytic index at all three time points, most notably during the first hour (28% increase relative to the untreated group; Figure 2D), although these differences were not statistically significant.

### 3.3. Effect of the Ca37 mAb Against C. auris in Galleria mellonella Animal Model

**Figure 3:**
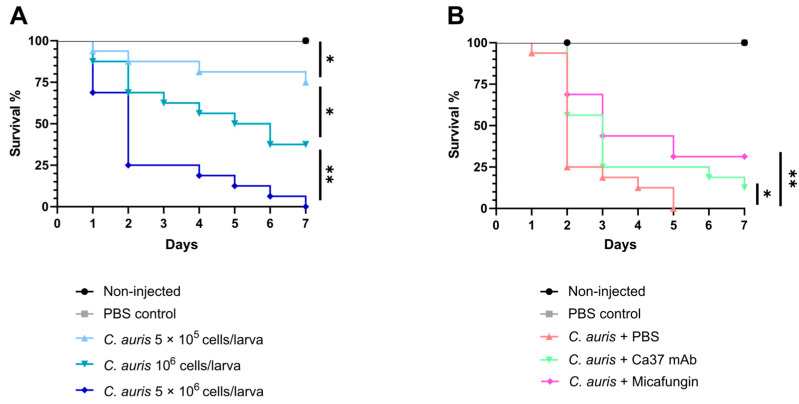
Dose–response and treatment efficacy of Ca37 mAb in *Galleria mellonella* larvae infected with *Candidozyma auris*. (**A**) Dose–response survival curves of *G. mellonella* larvae infected with *C. auris* CECT 13225 at three different inoculum doses (5 × 10^5^, 10^6^, and 5 × 10^6^ yeast cells per larva). (**B**) Survival curves of *G. mellonella* larvae infected with *C. auris* (5 × 10^6^ yeast cells per larva) followed by a second injection of PBS, 10 μg/mL Ca37 mAb, or 5 mg/mL micafungin. Non-injected and PBS-injected larvae were used as viability controls. Data shown are from a representative experiment (n = 16). Statistically significant differences are marked as * *p* < 0.05; ** *p* < 0.01 (Mantel–Cox test).

The protective effect of the antibody was evaluated in vivo using the *G. mellonella* animal model as an initial approach. First, the appropriate yeast inoculum was determined by testing doses of 5 × 10^5^, 1 × 10^6^, and 5 × 10^6^ *C. auris* CECT 13225 cells per larva. Although all doses induced significant mortality compared with uninfected controls, the highest dose was chosen because it consistently induced high mortality (Figure 3A). 

Once this optimal dose was selected, 5 × 10^6^ cells per larva were injected, followed by a second injection of either PBS (untreated control), 5 mg/kg micafungin (treatment control), or 10 µg/mL Ca37 mAb to evaluate antibody efficacy. Since the IgG1 isotype control had shown no inhibitory effect on fungal growth and appeared to promote it in some cases, it was excluded from subsequent experiments to minimise animal use. The Ca37 mAb significantly increased larval survival compared to the infected group receiving PBS, showing a protective effect comparable to that of micafungin (Figure 3B). The median survival was 2 days in the infected untreated group (PBS) and 3 days in larvae treated with either Ca37 mAb or micafungin. In contrast, both negative controls, larvae without injection and those injected twice with PBS, showed no mortality.

### 3.4. Effect of the Ca37 mAb Against C. auris in Mouse Animal Model

**Figure 4:**
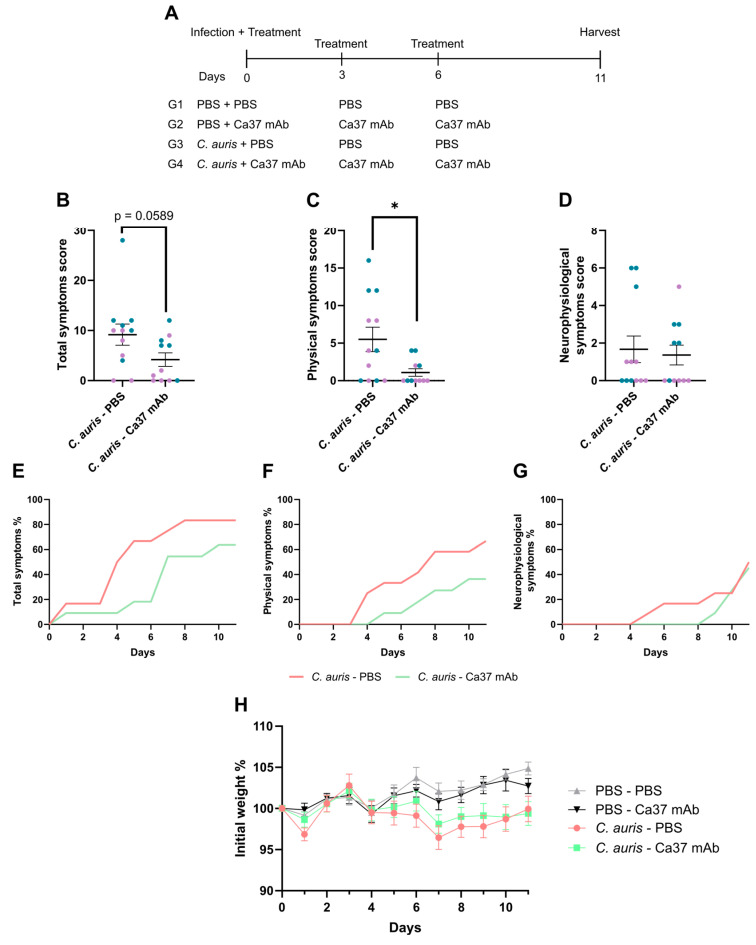
Study of the protective effect of Ca37 mAb on mouse weight and symptoms associated with *Candidozyma auris* infection. (**A**) The in vivo experimental design; Ca37 mAb was administered to reach 10 µg/mL in the mouse blood. Cumulative values of the recorded (**B**) total symptoms, (**C**) physical symptoms, and (**D**) neurophysiological symptoms over eleven days. Percentage of mice that developed (**E**) any symptoms, (**F**) physical symptoms, and (**G**) neurophysiological symptoms at any time during the experiment. (**H**) Body weight progression (% of initial weight) in the four groups: PBS—PBS (uninfected untreated), PBS—Ca37 mAb (uninfected treated), *C. auris*—PBS (infected untreated), and *C. auris*—Ca37 mAb (infected treated); each point represents the mean of the total group weight on each day. Immunocompetent mice were intravenously administered with *C. auris* CECT 13225 at a dose of 5 × 10^7^ yeast cells per animal. Blue represents male mice (n = 6 for *C. auris*—PBS and n = 5 for *C. auris*—Ca37 mAb), and purple represents female mice (n = 6 for *C. auris*—PBS and n = 6 for *C. auris*—Ca37 mAb). Data are presented as mean ± SEM. Statistically significant differences are marked as * *p* < 0.05 (two-tailed, unpaired, Student’s *t*-test).

Considering the results obtained using the invertebrate animal model, the study was extended to a murine systemic infection model. In the in vivo experimental design, summarised in Figure 4A, mice were distributed into four groups, with or without *C. auris* infection from the first day of the experiment, and received either PBS or Ca37 mAb as treatment. The uninfected group receiving Ca37 mAb served to assess its potential toxicity in mammals. Treatments were repeated on days three and six post-infection. For infections, based on our previous studies with *C. auris* CECT 13225, a dose of 5 × 10^7^ cells [13] was inoculated into 12 male and 12 female mice. No mortality occurred in any mouse group, except for one male in the infected-treated group, which died from anaesthesia-related complications.

Regarding the evaluation of the daily symptoms, based on Table S1, including weight-related scoring, the infected Ca37 mAb-treated group exhibited better overall well-being (Figure 4B), with a nearly significant difference in total symptom scores compared with the infected untreated group. In fact, when physical (excluding the weight lose) and neurophysiological symptoms were analysed separately, in both cases the treated group showed fewer symptoms, with statistically significant differences observed in the physical ones (Figure 4C,D). When symptomatology was analysed by sex, no significant differences were observed between male and female mice treated with the mAb. However, in the infected untreated group, a trend toward significance was noted when total symptoms were considered, with males exhibiting higher symptom scores than females (Figure S2).

The day-by-day analysis of total symptoms showed that, although they appeared from the beginning of the experiment in all *C. auris*-infected groups, their frequency was consistently lower in mice receiving mAb treatment (Figure 4E). Physical symptoms emerged at a similar time in both infected groups, with only a one-day delay in the treated animals; however, their progression was more pronounced in the untreated group, reaching 67%, whereas only 36% of the treated mice displayed such manifestations by the end of the observation period (Figure 4F). In contrast, the onset of neurophysiological symptoms was delayed by four days in the treated group, although by the final day of the experiment the same percentage of mice exhibited them (Figure 4G). Regarding weight loss (Figure 4H), the infected untreated group displayed a significant decrease in weight compared with non-infected groups from days 6 to 9, whereas the Ca37 mAb-treated group did not. Nevertheless, the weight of the infected treated group was non-significantly higher on most experimental days compared with the untreated group (Figure 4H). The uninfected groups, with or without mAb, showed no symptoms.

**Figure 5:**
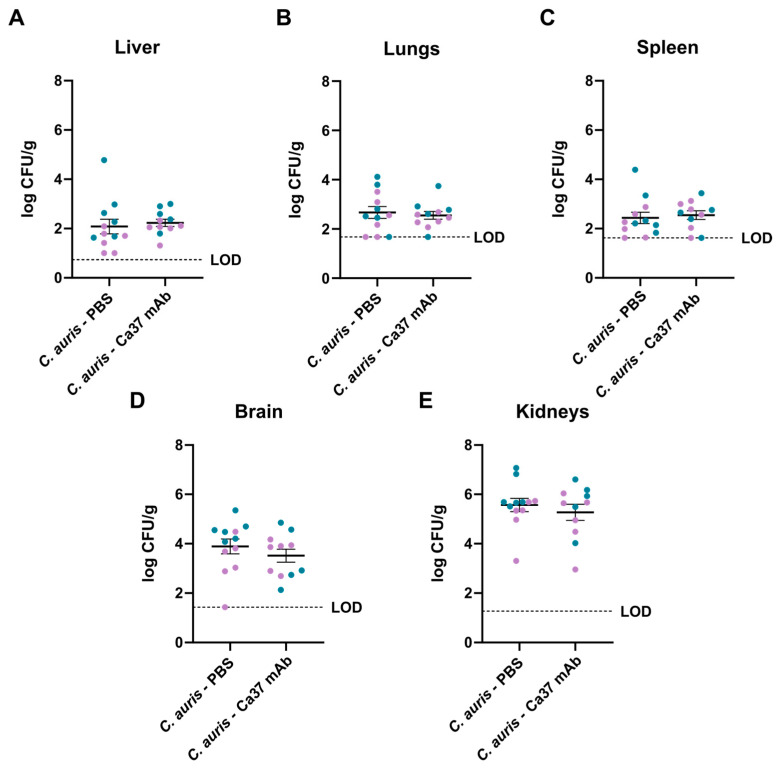
Fungal burden in organs of *Candidozyma auris*-infected mice from Ca37 mAb-treated and untreated groups. Data is represented as log CFU/g in (**A**) liver, (**B**) lungs, (**C**) spleen, (**D**) brain and (**E**) kidneys. The detection limit (LOD) for each organ is specified. Any data falling below this threshold, including values of zero, were treated as censored at the LOD. Immunocompetent mice were intravenously administered with *C. auris* CECT 13225 at a dose of 5 × 10^7^ yeast cells per animal. Blue represents male mice (n = 6 for *C. auris*—PBS and n = 5 for *C. auris*—Ca37 mAb), and purple represents female mice (n = 6 for *C. auris*—PBS and n = 6 for *C. auris*—Ca37 mAb). Data are presented as mean ± SEM. Data were analysed using a two-tailed unpaired Student’s *t* test.

After 11 days of experiment, mice were euthanised and, following organ collection, fungal load was quantified, and samples were histologically analysed to assess tissue involvement. In the uninfected groups, no fungal load was detected. Between the infected groups, although no statistically significant differences were observed in any organ in the group treated with the mAb compared to the untreated group, a slight reduction in fungal load was noted in the brain and kidneys, which also were the organs presenting the highest fungal burdens (Figure 5). When separating mice by sex, the differences between the infected groups were not significant. In addition, within the *C. auris*—PBS group, males exhibited higher fungal burdens in both the liver and the brain compared with females of the same group (Figure S3).

**Figure 6:**
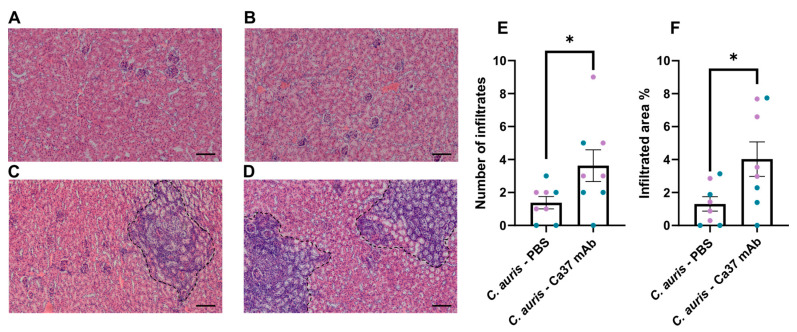
Effect of Ca37 mAb treatment on renal histopathology in *Candidozyma auris* infected mice. (**A**–**D**) Representative images of Haematoxylin –Eosin-stained female kidney sections from each experimental group after 11 days of infection. Scale bar: 100 μm: (**A**) PBS—PBS (uninfected untreated), (**B**) PBS—Ca37 mAb (uninfected treated), (**C**) *C. auris*—PBS (infected untreated), and (**D**) *C. auris*—Ca37 mAb (infected treated). Inflammatory infiltrates are marked by a dashed black line. (**E**) Quantification of inflammatory infiltrates per kidney field. (**F**) Percentage of infiltrated area per kidney section. Immunocompetent mice were intravenously administered with *C. auris* CECT 13225 at a dose of 5 × 10^7^ yeast cells per animal. Blue represents male mice (n = 4 for *C. auris*—PBS and n = 4 for *C. auris*—Ca37 mAb), and purple represents female mice (n = 4 for *C. auris*—PBS and n = 4 for *C. auris*—Ca37 mAb). Data are presented as mean ± SEM. Statistically significant differences are marked as * *p* < 0.05 (two-tailed, unpaired, Student’s *t* test).

To assess the histopathological impact of Ca37 mAb treatment during *C. auris* infection, kidney sections from each experimental group were examined by H&E staining (Figure 6A–D). Uninfected animals, either treated or untreated, showed normal renal architecture with no evidence of inflammation (Figure 6A,B). Infected mice receiving PBS exhibited focal inflammatory infiltrates and moderate tissue disruption (Figure 6C). Notably, infected mice treated with Ca37 mAb displayed increased inflammatory cell infiltration (Figure 6D), suggesting an enhanced immune response following antibody administration. Quantification of renal inflammation confirmed these observations. Both the number of inflammatory infiltrates per field (Figure 6E), and the percentage of infiltrated area per section (Figure 6F) were significantly higher in the Ca37 mAb-treated group compared to the infected untreated group. When analysing sexes separately, this difference remained significant only in females, while no significant difference was observed in males (Figure S4). On the other hand, a PAS staining showed that the infected untreated group contained more *C. auris*-like particles than the infected treated group (Figure S5C,D), consistent with the previously mentioned fungal burden. 

## 4. Discussion

In this study, we evaluated the antifungal potential of the Ca37 mAb against *C. auris*, an emerging multidrug-resistant pathogen associated with nosocomial outbreaks and high mortality [4]. The toxicity, limited efficacy, and emerging resistance of current antifungal therapies have intensified the search for alternative approaches, such as monoclonal antibody-based immunotherapy [25]. 

The Ca37 mAb generated by our group targeted the Adh of *C. albicans* [12], which is a well-characterised immunogenic protein with multiple roles in fungal pathogens. Several glycolytic enzymes, including Adh, function as moonlighting proteins in *Candida* spp., localising in the cell wall and contributing to virulence [10]. Their surface localisation facilitates interaction with host immune components and provides a target for antibody-mediated recognition. At least seven isoforms of *C. albicans* Adh have been identified, with Adh1 being the predominantly expressed variant and responsible for catalysing the conversion of ethanol to acetaldehyde, a reaction with implications for biofilm formation and morphogenesis [10].

In contrast, knowledge of Adh function in *C. auris* is limited. Recent studies suggest a distinct expression profile. Specifically, antifungal stress (e.g., pyrvinium pamoate treatment) induces upregulation of Adh1 and Adh5, consistent with a metabolic shift towards fermentation under mitochondrial dysfunction [26]. Moreover, Adh2 has been implicated in biofilm formation in antifungal resistant strains, suggesting functional specialisation across isoforms [27]. 

Given the relevance of this protein, the high sequence homology within the *Candida* and related genera, and its low similarity to human homologues (Table S2), this protein represents an attractive therapeutic target. Thus, the Ca37 mAb has previously shown inhibitory activity in vitro and protective effects in vivo against *C. albicans* infection in *G. mellonella* [12]. However, its effect on *Candida* species and other genera, including *C. auris*, was unknown.

Our data show that the Ca37 mAb is active in vitro not only against *C. albicans* [12] but also against *C. auris*, suggesting that surface-exposed Adh plays a role in fungal viability and is a target for antibody-mediated inhibition. Although our results do not conclusively demonstrate that Ca37 mAb specifically recognizes *C. auris* Adh, this protein was present in the mixture of CWAPs corresponding to the two spots detected by the mAb in the immunoproteomics assay.

In addition, the Ca37 mAb significantly inhibited *C. auris* growth at the same dose previously identified as optimal for *C. albicans* [12]. However, differences in growth inhibition between *C. albicans* and *C. auris* strains were observed. While growth inhibition in *C. albicans* ranged from 70% to 90% [12], inhibition among *C. auris* isolates was more variable, ranging from 46.5% to 83%. This difference between the species may reflect variations in the amino acid sequences of Adh, although the specific epitope recognised by Ca37 mAb has not yet been identified. The activity showed isolate-dependent variability, consistent with other findings using mAbs targeting other conserved fungal antigens, such as Phosphoglycerate kinase 1 (Pgk1) and trimannose carbohydrate (β-Man3), which also displayed differential effects across *C. auris* strains [28]. In our study, the isolate exhibiting the lowest inhibition (CECT 13226) corresponded to an aggregative phenotype. Although the aggregative phenotype has been linked to the evasion of phagocytosis [29], its potential to evade humoral immunity, such as antibodies, remains unclear. Further studies are needed to determine whether this phenotype contributes to reduced antibody accessibility or epitope masking.

The Ca37 mAb also enhanced phagocytosis by murine macrophages, suggesting a dual mechanism of action combining direct antifungal activity and immune-mediated mechanisms. This dual mechanism is consistent with previous reports on antifungal mAbs against *C. auris* and other fungi [28,30,31]. 

In vivo, similar to the effect observed with *C. albicans* [12], the Ca37 mAb conferred protection to *G. mellonella* larvae infected with *C. auris*, as evidenced by a statistically significant increase in survival compared to untreated control. These findings indicate that the Ca37 mAb exerts a protective effect in *G. mellonella* against both species. 

Finally, the Ca37 mAb was evaluated in an immunocompetent murine model to confirm its efficacy in a mammalian host. Among infected mice, untreated animals showed a greater, although not statistically significant weight loss compared with those treated with the Ca37 mAb. Moreover, regarding animal welfare, whereas no clinical symptoms were observed in uninfected mice, those infected but treated with the Ca37 mAb exhibited fewer clinical signs and delayed onset of both physical and neurophysiological symptoms, compared to the infected untreated group, indicating a potential protective effect of the antibody on overall clinical condition. 

No significant differences in fungal CFU counts were detected across the organs analysed between the infected groups, but the reduction in fungal loads in kidneys and brain (the two organs showing the highest levels of fungal burden) of treated animals warrants further investigation, including the use of other different mAb doses. It is important to note, however, that fungal burden was only assessed at the experimental endpoint, and the fungal load at earlier time points, particularly during antibody administration, remains unknown. Furthermore, the pharmacodynamics of the mAb are unknown. Indeed, other studies evaluating anti-*C. auris* mAbs in murine models have reported reductions in fungal burden as early as day 3, 4, or 7 post-infection in immunosuppressed mice [28,30,31]. Regarding sex-related differences, in the infected-untreated group, fungal loads in the brain and liver were higher in males than in females. Similarly, males exhibited more pronounced symptoms in general. This could be related to the stronger immunological responses previously reported in females compared with males [32]. However, few studies have included both sexes in systemic *C. auris* infections in mice, and most research has not specifically addressed sex-related differences. 

Histological analysis of the kidneys revealed no detectable abnormalities in both uninfected groups (PBS—PBS and PBS—Ca37 mAb), supporting the safety profile of Ca37 mAb. However, the infected Ca37 mAb-treated group displayed a more extensive immune infiltration and a larger infiltrated area in the kidneys than infected untreated mice, potentially indicating enhanced immune recognition of *C. auris* facilitated by the mAb. Notably, while this enhanced immune response did not result in a significant reduction in fungal burden, it did correlate with improved clinical condition. These findings suggest that Ca37 mAb may contribute to host defence by enhancing opsonisation and/or immune recruitment rather than exerting a direct fungicidal effect in vivo, although the underlying processes remain to be elucidated. This effect could be particularly relevant in the context of *C. auris*, a pathogen known for its ability to evade immune recognition [33]. In the case of other described anti-*C. auris* mAbs, these showed reduction in fungal burden accompanied by decreased immune cell infiltration [30] or reduced serum levels of inflammatory markers [28], accompanied by a reduction in fungal burden. However, models are completely different, as they used immunosuppressed mice and studied the effect at shorter period of infection. 

Despite the results presented, this study has also some limitations such as the lack of assessment of the synergistic effect with conventional antifungal drugs, as Ca37 demonstrated in vitro with fluconazole and micafungin against *C. albicans* [12]. Similarly, other monoclonal antibodies have shown activity against biofilms in vitro [28,30] suggesting that evaluating Ca37 mAb against *C. auris* biofilms could also be of considerable interest. Moreover, including isolates from different *C. auris* clades would be valuable. Future studies addressing these aspects will help to better define the therapeutic potential of Ca37 mAb.

Therefore, the findings of this study suggest that Ca37 mAb targets a cell wall-associated antigen involved in host–pathogen interactions and highlight its potential as a candidate for immunotherapy against *C. auris*. The observed in vitro inhibition of *C. auris*, enhanced phagocytosis by murine macrophages, and protective effect in *G. mellonella* further support its potential. Nevertheless, optimising dosing regimens, treatment timing, and exploring potential synergistic effects with existing antifungals will be critical to maximise efficacy in mammalian models. Although improvements in health status and a greater immune response against *C. auris* were observed in the treated murine model, additional studies are needed to refine this approach and validate its translational relevance.

## Data Availability

The data of the protein identification has been deposited at ProteomeXchange Consortium via the PRIDE partner repository with the dataset identifier PXD070229.

## Supplementary Materials

The following supporting information can be downloaded at: https://www.mdpi.com/article/10.3390/jof11120864/s1. Table S1: System for scoring used to monitor the mice, assessing both the presence and severity of relevant symptoms; Table S2: Comparison of *Candida albicans* Adh protein sequence identity among different fungal species, human and mice; Figure S1: Effect of Ca37 mAb on the growth curve of *Candidozyma auris* CECT 13225 in SDB medium; Figure S2: Effect of Ca37 mAb on mice weight, physical and neurological symptoms, separated by sex; Figure S3: Fungal burden in different organs of *Candidozyma auris* infected mice, separated by sex; Figure S4: Effect of Ca37 mAb in renal histopathology of *Candidozyma auris* infected mice, separated by sex; Figure S5: PAS-stained renal sections illustrating the effect of Ca37 mAb treatment in *Candidozyma auris*-infected mice.

## Abbreviations

The following abbreviations are used in this manuscript:

2D-WB	Two-dimensional electrophoresis followed by Western blot
Adh	Alcohol dehydrogenase
CECT	Spanish Culture Type Collection
CFUs	Colony Forming Units
CWAP	Cell wall-associated protein
H&E	Haematoxylin–Eosin
LC-MS/MS	Liquid Chromatography–Mass Spectrometry
LOD	Limit of detection
mAb	Monoclonal antibody
MIC	Minimal inhibitory concentration
MOI	Multiplicity of Infection
PBS	Phosphate-Buffered Saline
PMSF	Phenylmethylsulfonyl fluoride
SDA	Sabouraud Dextrose Agar
SDB	Sabouraud Dextrose Broth
TBSM	Tris-Buffered Saline Milk
WB	Western blot
WHO	World Health Organization

## References

[1] Satoh K., Makimura K., Hasumi Y., Nishiyama Y., Uchida K., Yamaguchi H. (2009). *Candida auris* Sp. Nov., a Novel Ascomycetous Yeast Isolated from the External Ear Canal of an Inpatient in a Japanese Hospital. Microbiol. Immunol..

[2] Liu F., Hu Z.D., Zhao X.M., Zhao W.N., Feng Z.X., Yurkov A., Alwasel S., Boekhout T., Bensch K., Hui F.L. (2024). Phylogenomic Analysis of the *Candida auris*- *Candida haemuli* Clade and Related Taxa in the Metschnikowiaceae, and Proposal of Thirteen New Genera, Fifty-Five New Combinations and Nine New Species. Persoonia Mol. Phylogeny Evol. Fungi.

[3] WHO (2022). WHO Fungal Priority Pathogens List to Guide Research, Development and Public Health Action.

[4] Jacobs S.E., Jacobs J.L., Dennis E.K., Taimur S., Rana M., Patel D., Gitman M., Patel G., Schaefer S., Iyer K. (2022). *Candida auris* Pan-Drug-Resistant to Four Classes of Antifungal Agents. Antimicrob. Agents Chemother..

[5] Eyre D.W., Sheppard A.E., Madder H., Moir I., Moroney R., Quan T.P., Griffiths D., George S., Butcher L., Morgan M. (2018). A *Candida auris* Outbreak and Its Control in an Intensive Care Setting. N. Engl. J. Med..

[6] Proctor D.M., Dangana T., Sexton D.J., Fukuda C., Yelin R.D., Stanley M., Bell P.B., Baskaran S., Deming C., Chen Q. (2021). Integrated Genomic, Epidemiologic Investigation of *Candida auris* Skin Colonization in a Skilled Nursing Facility. Nat. Med..

[7] Kordalewska M., Perlin D.S. (2019). Identification of Drug Resistant *Candida auris*. Front. Microbiol..

[8] Lockhart S.R., Etienne K.A., Vallabhaneni S., Farooqi J., Chowdhary A., Govender N.P., Colombo A.L., Calvo B., Cuomo C.A., Desjardins C.A. (2017). Simultaneous Emergence of Multidrug-Resistant *Candida auris* on 3 Continents Confirmed by Whole-Genome Sequencing and Epidemiological Analyses. Clin. Infect. Dis..

[9] Ulrich S., Ebel F. (2020). Monoclonal Antibodies as Tools to Combat Fungal Infections. J. Fungi.

[10] Wang Z., Zhang Q., Zhang H., Lu Y. (2024). Roles of Alcohol Dehydrogenase 1 in the Biological Activities of *Candida albicans*. Crit. Rev. Microbiol..

[11] Crowe J.D., Sievwright I.K., Auld G.C., Moore N.R., Gow N.A.R., Booth N.A. (2003). *Candida albicans* Binds Human Plasminogen: Identification of Eight Plasminogen-Binding Proteins. Mol. Microbiol..

[12] Antoran A., Aparicio-Fernandez L., Pellon A., Buldain I., Martin-Souto L., Rementeria A., Ghannoum M.A., Fuchs B.B., Mylonakis E., Hernando F.L. (2020). The Monoclonal Antibody Ca37, Developed against *Candida albicans* Alcohol Dehydrogenase, Inhibits the Yeast in Vitro and in Vivo. Sci. Rep..

[13] Areitio M., Antoran A., Rodriguez-Erenaga O., Aparicio-Fernandez L., Martin-Souto L., Buldain I., Zaldibar B., Ruiz-Gaitan A., Pemán J., Rementeria A. (2024). Identification of the Most Immunoreactive Antigens of *Candida auris* to IgGs from Systemic Infections in Mice. J. Proteome Res..

[14] Pitarch A., Sánchez M., Nombela C., Gil C. (2002). Sequential Fractionation and Two-Dimensional Gel Analysis Unravels the Complexity of the Dimorphic Fungus *Candida albicans* Cell Wall Proteome. Mol. Cell Proteom..

[15] Perez-riv Y., Bandla C., Kundu D.J., Kamatchinathan S., Bai J., Hewapathirana S., John N.S., Prakash A., Walzer M., Wang S. (2025). The PRIDE Database at 20 Years: 2025 Update. Nucleic Acids Res..

[16] Magliani W., Conti S., Bernardis F.D., Gerloni M., Bertolotti D., Mozzoni P., Cassone A., Polonelli L. (1997). Therapeutic Potential of Antiidiotypic Single Chain Antibodies with Yeast Killer Toxin Activity. Nat. Biotechnol..

[17] Sadavarte R.H., Ghosh R. (2014). A Thermal-Cycling Method for Disaggregating Monoclonal Antibody Oligomers. J. Pharm. Sci..

[18] Fuchs B.B., O’Brien E., Khoury J.B.E., Mylonakis E. (2010). Methods for Using *Galleria mellonella* as a Model Host to Study Fungal Pathogenesis. Virulence.

[19] Hager C.L., Larkin E.L., Long L.A., Ghannoum M.A. (2018). Evaluation of the Efficacy of Rezafungin, a Novel Echinocandin, in the Treatment of Disseminated *Candida auris* Infection Using an Immunocompromised Mouse Model. J. Antimicrob. Chemother..

[20] Fuchs B.B., Li Y., Li D., Johnston T., Hendricks G., Li G., Rajamuthiah R., Mylonakis E. (2016). Micafungin Elicits an Immunomodulatory Effect in *Galleria mellonella* and Mice. Mycopathologia.

[21] National Centre for the Replacement Refinement & Reduction of Animals in Research Blood Sampling: Mouse. https://nc3rs.org.uk/3rs-resource-library/blood-sampling/blood-sampling-mouse.

[22] Bancroft J.D., Gamble M. (2002). Theory and Practice of Histological Techniques.

[23] Suvarna S.K., Layton C., Bancroft J.D. (2018). Bancroft’s Theory and Practice of Histological Techniques.

[24] Areitio M., Rodriguez-Erenaga O., Aparicio-Fernandez L., Abio-Dorronsoro L., Martin-Souto L., Perez-Cuesta U., Buldain I., Zaldibar B., Ruiz-Gaitan A., Pemán P. (2025). The Oxidative Stress-Related Peroxiredoxin Tsa1b of *Candidozyma* (*Candida*) *auris* Contributes to Virulence and Infection. Microbiol. Res..

[25] Boniche C., Rossi S.A., Kischkel B., Barbalho F.V., Moura Á.N.D., Nosanchuk J.D., Travassos L.R., Taborda C.P. (2020). Immunotherapy against Systemic Fungal Infections Based on Monoclonal Antibodies. J. Fungi.

[26] Simm C., Weerasinghe H., Thomas D.R., Harrison P.F., Newton H.J., Beilharz T.H., Traven A. (2022). Disruption of Iron Homeostasis and Mitochondrial Metabolism Are Promising Targets to Inhibit *Candida auris*. Microbiol. Spectr..

[27] Zhou W., Li X., Lin Y., Yan W., Jiang S., Huang X., Yang X., Qiao D., Li N. (2021). A Comparative Transcriptome Between Anti-Drug Sensitive and Resistant *Candida auris* in China. Front. Microbiol..

[28] Rosario-Colon J., Eberle K., Xin H. (2025). Monoclonal Antibodies Targeting *Candida* Disrupt Biofilms and Inhibit Growth across Global Clinical Isolates. iScience.

[29] Pelletier C., Shaw S., Alsayegh S., Brown A.J.P., Lorenz A. (2024). *Candida auris* Undergoes Adhesin-Dependent and -Independent Cellular Aggregation. PLoS Pathog..

[30] Singh S., Barbarino A., Youssef E.G., Coleman D., Gebremariam T., Ibrahim A.S. (2023). Protective Efficacy of Anti-Hyr1p Monoclonal Antibody against Systemic Candidiasis Due to Multi-Drug-Resistant *Candida auris*. J. Fungi.

[31] Rudkin F.M., Raziunaite I., Workman H., Essono S., Belmonte R., MacCallum D.M., Johnson E.M., Silva L.M., Palma A.S., Feizi T. (2018). Single Human B Cell-Derived Monoclonal Anti-*Candida* Antibodies Enhance Phagocytosis and Protect against Disseminated Candidiasis. Nat. Commun..

[32] Lu R.J., Taylor S., Contrepois K., Kim M., Bravo J.I., Ellenberger M., Sampathkumar N.K., Benayoun B.A. (2021). Multi-Omic Profiling of Primary Mouse Neutrophils Predicts a Pattern of Sex- and Age-Related Functional Regulation. Nat. Aging.

[33] Wang Y., Zou Y., Chen X., Li H., Yin Z., Zhang B., Xu Y., Zhang Y., Zhang R., Huang X. (2022). Innate Immune Responses against the Fungal Pathogen *Candida auris*. Nat. Commun..

