# Supplementary material for: Evaluation of the Ca37 Monoclonal Antibody Targeting Alcohol Dehydrogenase Against Candidozyma auris (Candida auris) In Vitro and In Vivo
Source: J Fungi (Basel). 2025 Dec 5;11(12):864. doi: 10.3390/jof11120864 (PMC12733945; doi:10.3390/jof11120864)
Supplement: Supplementary file 1 [file jof-11-00864-s001.zip › jof-3932865-supplementary.pdf]

Journal of Fungi

MDPI

Supplementary material

Evaluation of the Ca37 monoclonal antibody targeting alcohol dehydrogenase against *Candidozyma auris* (*Candida auris*) in vitro and in vivo

Oier Rodriguez-Erenaga[1], Maialen Areitio[1,2], Lucia Abio-Dorronsoro[1], Nahia Cazalis-Bereicua[1], Leire Aparicio-Fernandez[1,3], Leire Martin-Souto[1], Idoia Buldain[3], Beñat Zaldibar[4], Aitor Rementeria[1]*, Aitziber Antoran[1]* and Andoni Ramirez-Garcia[1]

[1] MicrobiomicsEHU Research Group, Department of Immunology, Microbiology and Parasitology, University of the Basque Country (UPV/EHU), Leioa, Spain
[2] Section of Immunology, Vetsuisse Faculty, and Institute of Experimental Immunology, University of Zürich (UZH), Zurich, Switzerland
[3] MicrobiomicsEHU Research Group, Department of Immunology, Microbiology and Parasitology, University of the Basque Country (UPV/EHU), Vitoria-Gasteiz, Spain
[4] CBET Research Group, Department of Zoology and Animal Cell Biology, Faculty of Science and Technology, Research Centre for Experimental Marine Biology and Biotechnology PIE, University of the Basque Country (UPV/EHU), 48940 Leioa, Spain

* Correspondence: aitziber.antoran@ehu.eus (A.A); Tel.: +34 94 601 5407; aitor.rementeria@ehu.eus (A.R); Tel.: +34 94 601 5964

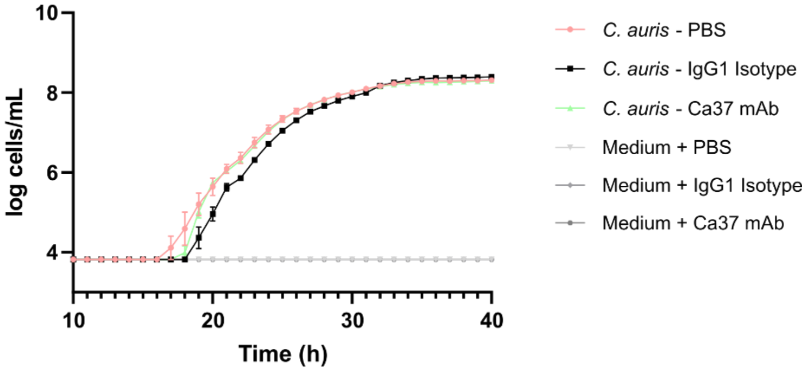

Figure S1. Effect of Ca37 mAb on the growth curve of *Candidozyma auris* CECT 13225 in SDB medium. Cultures were initiated at the same starting density (1.5 × 10^3 cells/mL) and incubated at 37 °C. Growth was monitored by measuring OD_{600} every 10 minutes after shaking the plate for 1 minute, and hourly data points are shown. OD values were converted to $\log_{10}$ cells/mL us-ing a calibration curve. Three conditions were compared: Ca37 mAb (10 µg/mL), IgG1 isotype control (10 µg/mL), and untreated control (PBS). Data are presented as mean ± SEM from three independent biological replicates. The medium containing each treatment is also shown.

Journal of Fungi

MDPI

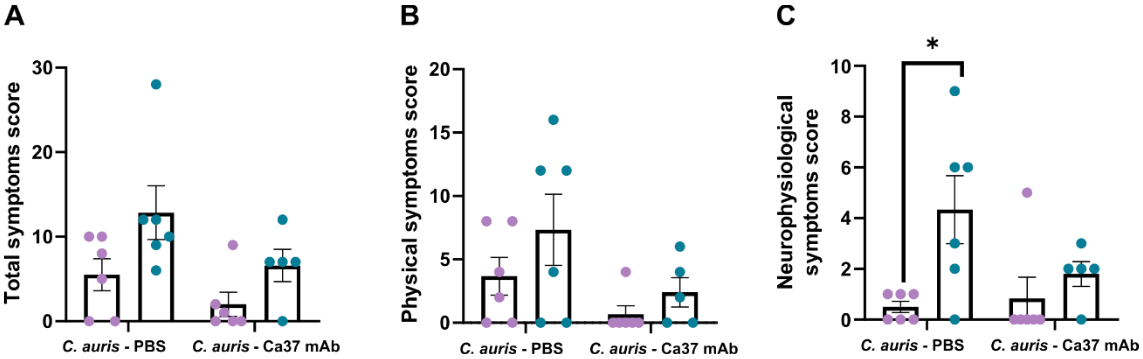

Figure S2. Study of the protective effect of the Ca37 mAb on mouse weight and symptoms associated with *Candidozyma auris* infection, shown separately for males and females. Cumulative values of (A) total recorded symptoms, (B) physical symptoms, and (C) neurophysiological symptoms over eleven days. Blue represents male mice (n = 6 for *C. auris* – PBS and n = 5 for *C. auris* – Ca37 mAb), and purple represents female mice (n = 6 for *C. auris* – PBS and n = 6 for *C. auris* – Ca37 mAb). Data are presented as mean ± SEM. Statistically significant differences are marked as *p < 0.05 (two-tailed, unpaired, Student's *t* test).

Journal of Fungi

MDPI

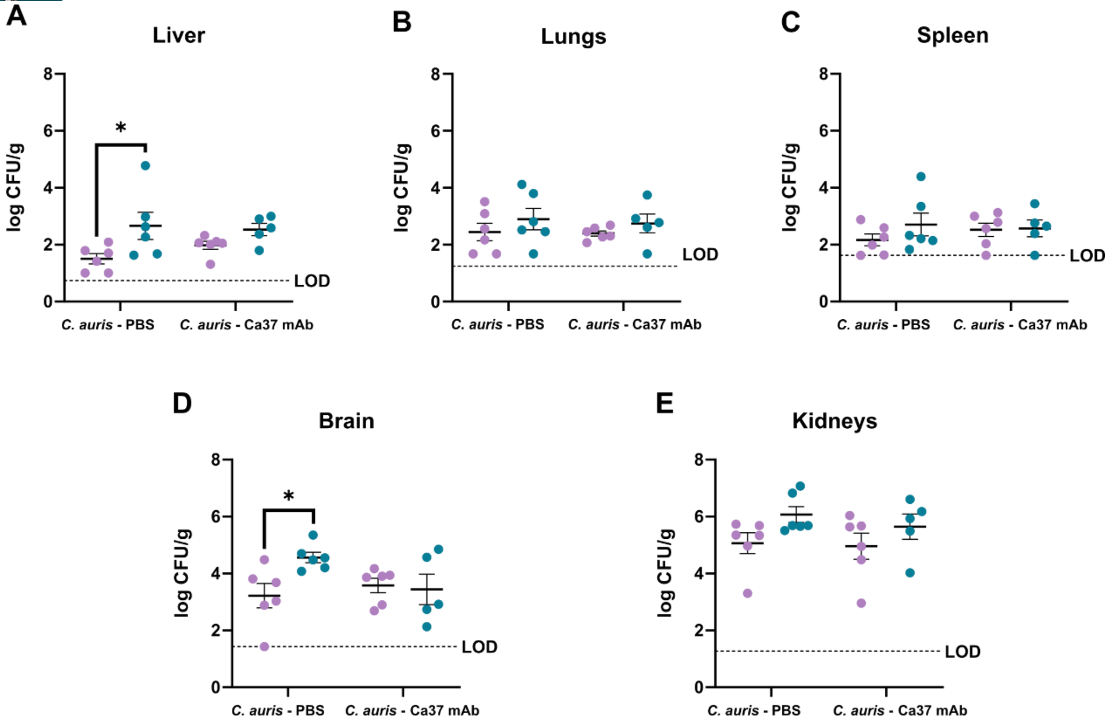

Figure S3. Fungal burden in organs of *Candidozyma auris*-infected mice from Ca37 mAb-treated and untreated groups, shown separately for males and females. Data are represented as log CFU/g in (A) liver, (B) lungs, (C) spleen, (D) brain, and (E) kidneys. The detection limit (LOD) for each organ is specified. Any data falling below this threshold, including values of zero, were treated as censored at the LOD. Immunocompetent mice were intravenously administered with *C. auris* CECT 13225 at a dose of 5×10^7 yeast cells per animal. Blue represents male mice (n = 6 for *C. auris* – PBS and n = 5 for *C. auris* – Ca37 mAb), and purple represents female mice (n = 6 for *C. auris* – PBS and n = 6 for *C. auris* – Ca37 mAb). Data are presented as mean ± SEM. Statistically significant differences are marked as *p < 0.05 (two-tailed, unpaired, Student's *t* test).

Journal of Fungi

MDPI

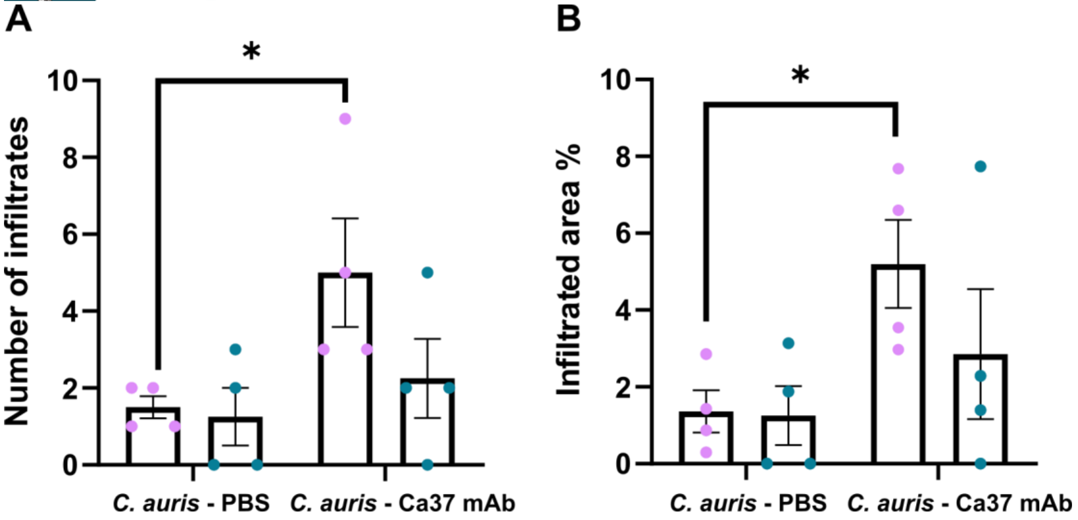

Figure S4. Effect of Ca37 mAb treatment on renal histopathology in *Candidozyma auris* infected mice, shown separately for males and females. (A) Quantification of inflammatory infiltrates per kidney field. (B) Percentage of infiltrated area per kidney section. Immunocompetent mice were intravenously administered with *C. auris* CECT 13225 at a dose of 5×10^7 yeast cells per animal. Blue represents male mice (n = 4 for *C. auris* – PBS and n = 4 for *C. auris* – Ca37 mAb), and purple represents female mice (n = 4 for *C. auris* – PBS and n = 4 for *C. auris* – Ca37 mAb). Data are presented as mean ± SEM. Statistically significant differences are marked as *$p < 0.05$ (two-tailed, unpaired, Student's *t* test).

Journal of Fungi

MDPI

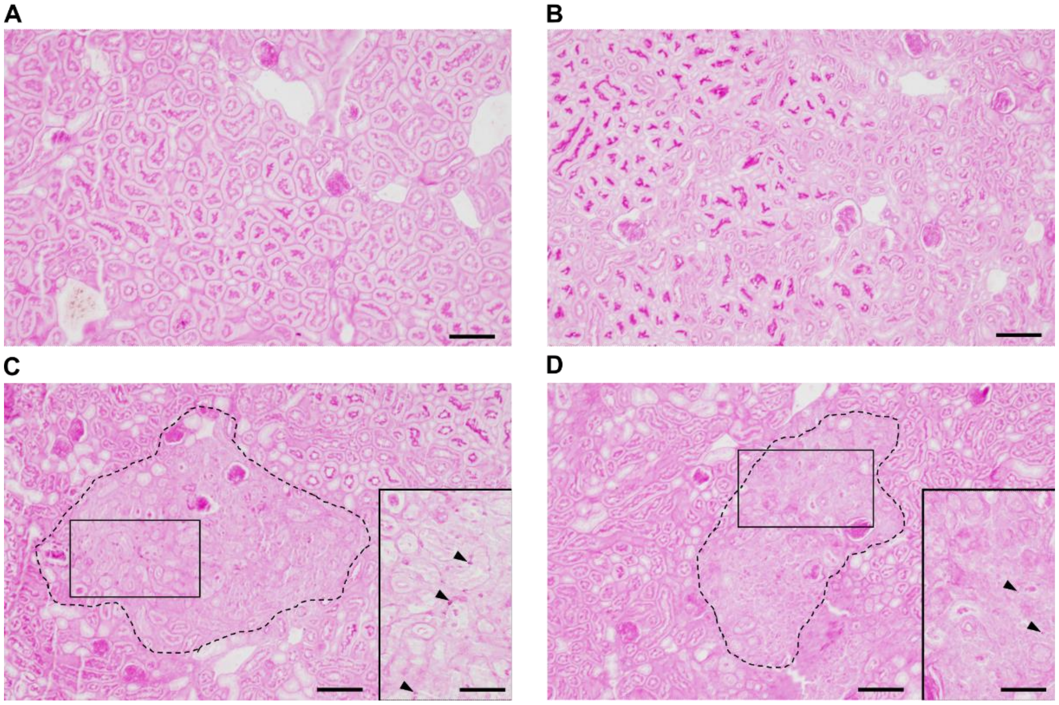

Figure S5. PAS-stained renal sections illustrating the effect of Ca37 mAb treatment in *Candidozyma auris*-infected mice. Representative images of PAS-stained female kidney sections from each experimental group after 11 days of infection to visualise PAS-positive fungal structures. Scale bar: 100 µm and 25 µm (insets): (A) PBS - PBS (uninfected untreated), (B) PBS - Ca37 mAb (uninfected treated), (C) *C. auris* - PBS (infected untreated), and (D) *C. auris* - Ca37 mAb (infected treated). Dashed black lines mark inflammatory infiltrates, black squares indicate the corresponding insets in panels C and D, and black triangles denote PAS-positive elements compatible with fungal cells.

Journal of Fungi

MDPI

Table S1. System for scoring used to monitor mice, assessing both the presence and severity of relevant symptoms. The table displays general and specific symptoms along with the scores assigned to each (ranging from 1 to 4).

General Symptom	Specific symptom	Value (1-4)
Body weight	Loss between 5-10%	1
	Loss between 10-20%	2
	Loss between 20-25%	3
	Loss equal to or greater than 25%	4
Transient discomfort after injection		1
Abnormal postures	Hunched abdomen	2
	Stretching of the body	2
Weakness or paralysis of the limbs		4
Skin alteration	Changes in skin consistency	1
	Ruffled fur	2
Stool appearance	Soft	1
	Diarrhoea	2
	Blood in stool	3
	Diarrhoea > 48 hours	4
Feeding and drinking	Transient anorexia after injection	1
	Recurrent anorexia	2
	Not drinking	3
Breathing	Tachypnoea	1
	Dyspnoea	2
	Severe dyspnoea	4
Neurological disturbances	Head bobbing	1
	Leaning to one side	1
	Ataxia	2
	Jumping	2
	Complete loss of balance	4
Behaviour	Transient lethargy after injection	1
	Stereotypies	1
	Moderate change in behaviour and/or withdrawal from peers	2
	Persistent lethargy	3
	Reacts violently/vocalisation	3
Physical parameters	Distension of the abdomen	2
	Cachexia	4
	20% increase in body circumference	4

Journal of Fungi

MDPI

Table S2. Comparison of *Candida albicans* Adh protein sequence identity among different fungal species, human and mice (https://blast.ncbi.nlm.nih.gov/Blast.cgi).

**Species**	**Identity % respect to *C. albic*ans Adh**
C. auris	80
C. parapsilosis	85
N. glabratus	73
Homo sapiens	32
Mus musculus	29
